# Deep learning enabled multi-organ segmentation of mouse embryos

**DOI:** 10.1242/bio.059698

**Published:** 2023-02-21

**Authors:** S. M. Rolfe, S. M. Whikehart, A. M. Maga

**Affiliations:** ^1^ Center for Developmental Biology and Regenerative Medicine, Seattle Children's Research Institute, Seattle, WA 98101, USA; ^2^ Department of Pediatrics, University of Washington, Seattle, WA 98105, USA

**Keywords:** Segmentation, Deep learning, Embryo, Micro-CT, Mouse, Automated

## Abstract

The International Mouse Phenotyping Consortium (IMPC) has generated a large repository of three-dimensional (3D) imaging data from mouse embryos, providing a rich resource for investigating phenotype/genotype interactions. While the data is freely available, the computing resources and human effort required to segment these images for analysis of individual structures can create a significant hurdle for research. In this paper, we present an open source, deep learning-enabled tool, Mouse Embryo Multi-Organ Segmentation (MEMOS), that estimates a segmentation of 50 anatomical structures with a support for manually reviewing, editing, and analyzing the estimated segmentation in a single application. MEMOS is implemented as an extension on the 3D Slicer platform and is designed to be accessible to researchers without coding experience. We validate the performance of MEMOS-generated segmentations through comparison to state-of-the-art atlas-based segmentation and quantification of previously reported anatomical abnormalities in a *Cbx4* knockout strain.

This article has an associated First Person interview with the first author of the paper.

## Teaser

**Summary:** We present a new open source, deep learning-enabled tool, Mouse Embryo Multi-Organ Segmentation (MEMOS), to estimate the segmentation of 50 anatomical structures from microCT scans of embryonic mice.

## INTRODUCTION

Knockout Mouse Phenotyping Program (KOMP2), a Common Fund and trans-National Institute of Health project that includes 18 institutes and centers, was initiated with the goal of providing a comprehensive and public resource for exploring genotype/phenotype interactions in null mutant mice (Dickinson et al., 2016). KOMP2 is a part of the IMPC, an international effort to identify the function of every protein coding gene in the mouse genome. Currently 7022 of the 23,000 targeted genes have been evaluated. As part of the standardized phenotyping pipeline, high-resolution 3D micro-CT fetal imaging is collected for sub-viable and lethal strains. So far, 267 lines at E14.5/E15.5 with an average of six embryos, have been scanned and new lines are continually added. The high-resolution micro-CT imaging data, along with large sample of wild-type control group provides the capability to detect even subtle differences in organ size and shape in knockout strains (Dickinson et al., 2016; Wong et al., 2012).

3D imaged fetal specimens are amenable to quantitative phenotyping by measuring the organ sizes and shapes, and their deviation from the normative sample can be a strong indicator of the role a particular knocked-out gene may play in normal development of the organ. However, segmentation of whole-body micro-CT scan images can be a challenging problem due to variation in shape across specimens, the low contrast of soft tissue, and complexity of the three-dimensional (3D) organ shape. The current best practice is to have an expert manually add these labels, which is time consuming and prone to variability across time periods, individuals, and datasets. Manual segmentation of the 50 anatomical structures necessary to generate a fetal atlas can take up to a few hundred hours to complete, e.g. Wong et al. (2012) cited approximately 400 manhours to derive the E15 mouse fetal atlas segmentation. Thanks to the improvement in semi-automated interactive segmentation tools, an expert can probably complete similar tasks a little faster, but in our experience, it is still necessary to commit anywhere from 40-100 h to fully segment a single E15 mouse scan from the IMPC collection. While this may be feasible for analysis of individuals or small groups, the increasing availability of 3D imaging in big data applications over the last decade has driven a need for automated pipelines that can support the increased throughput necessary to quantify normal variation and detect small-scale differences in phenotype.

Atlas-based image registration has been long used as a method to automate segmentation. In these methods, one (or more) reference images are chosen to serve as an index for a population. Segments or other annotations placed on a reference image are transferred to individual specimen via dense, deformable image registration that provide voxel correspondences between the reference and subject (Park et al., 2003; Xu et al., 2015). Atlas-based methods for segmentation (ABM) have now become widely used to improve both the speed and accuracy of segmentations, including application to the fetal mouse images from the KOMP2 dataset where it was demonstrated to be sensitive enough to identify abnormalities in knockout strains (Horner et al., 2021; Van Der Heyden et al., 2019; Wong et al., 2012). While the gains provided by ABM are meaningful, these methods are highly computationally intensive and typically require specialized, high-cost compute clusters that take significant time and expertise to maintain, creating another barrier to open data access and repeatable results. These methods can also show a bias towards normal anatomy as large-scale deformations may be limited by the robustness and computational time of the image transform. This presents a challenge for segmenting highly deformable internal organs with large-scale anatomical variation between individuals. ABM can also be sensitive to the selection of the atlas or multiple atlases (Heckemann et al., 2006; Van Der Heyden et al., 2019).

Recently, deep networks have become a competitive method for automating segmentation in medical and biological imaging applications, with performance exceeding state-of-the-art ABM and demonstrated advantages in computational requirements (Hatamizadeh et al., 2022; Schoppe et al., 2020; Tang et al., 2021). Deep learning methods also have the potential for performance improvement in regions where anatomy is highly variable and may not be well modelled by a reference segmentation, as is required by ABM. While the training process to generate a segmentation model is computationally intensive, requiring several hours and specialized hardware, once trained, a deployed network can be comparatively lightweight. Inference of an estimated segmentation for a new specimen typically requires a fraction of the computational time and is possible to run on a standard desktop computer.

**Fig. 1.**
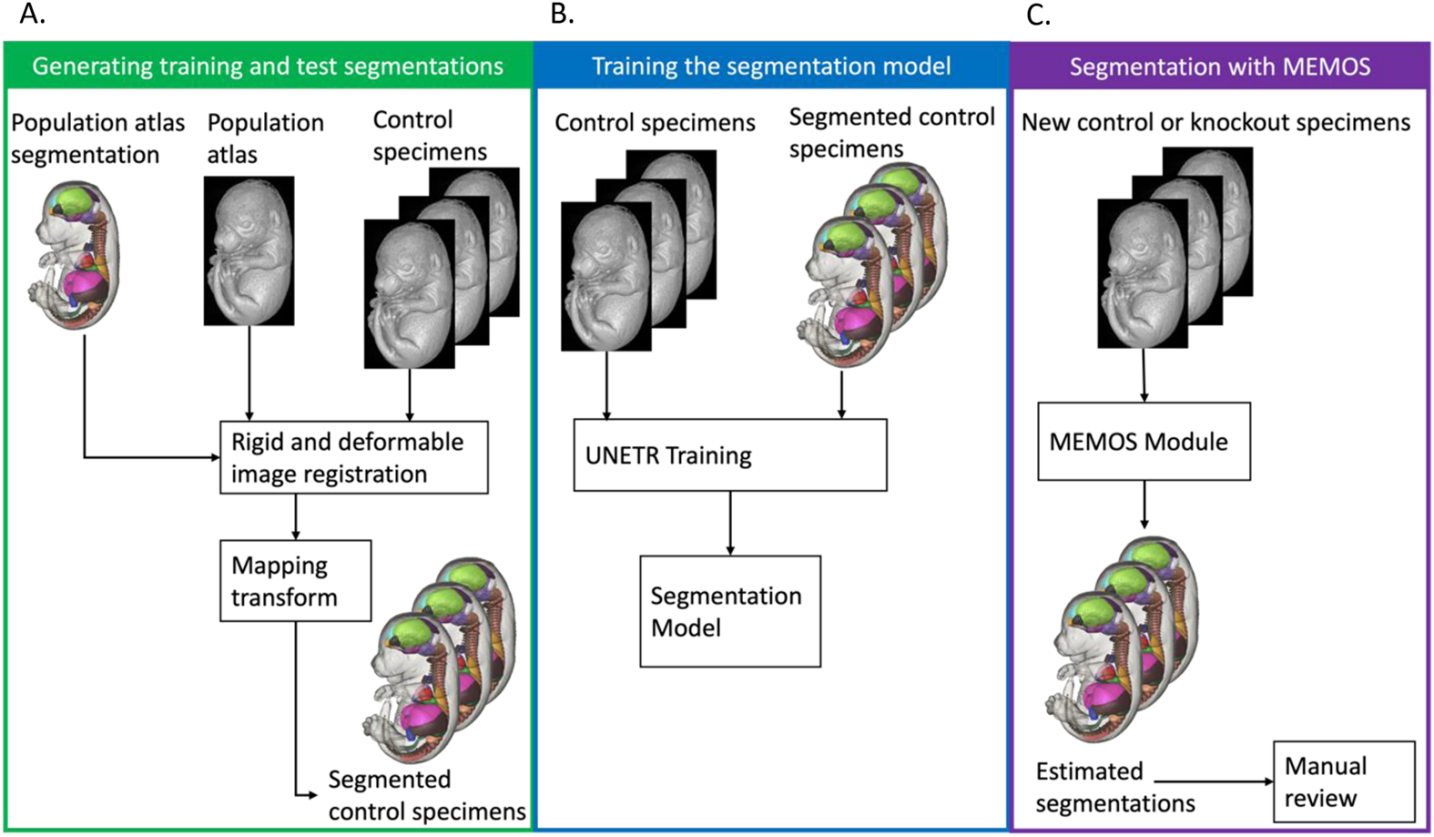
**Workflow diagram for development and deployment of the MEMOS segmentation module.** The three phases of this project included (A) creating labeled training data from the KOMP2 E15 population atlas and labels using ABM, (B) training a segmentation model on the ABM generated labels, and (C) deploying the fully trained model in the MEMOS extension to the 3D Slicer application.

In this paper we present Mouse Embryo Multi-Organ Segmentation (MEMOS), a new deep learning-powered tool for segmentation of fetal mice, with the goal of supporting open-access analysis of imaging data collected as part of the IMPC's KOMP project. MEMOS is provided as a general tool to be used in a semi-supervised pipeline for fast and highly accurate segmentations or fine-tuned for customized applications. The development of MEMOS is comprised of three main tasks: generating ‘gold-standard’ segmentations for training, training the segmentation model, and deployment of the model in the opensource, user-friendly MEMOS module. This workflow is summarized in Fig. 1.

## RESULTS

### Segmentation performance

We implemented the deep leaning network for MEMOS using PyTorch and the Medical Open Network for Artificial Intelligence (MONAI) libraries (Diaz-Pinto et al., 2022 preprint). MONAI is an open-source framework for deep learning customized to work with healthcare imaging, including image input and output functions, data processing and image transformation pipelines. Since the application in this work was non-clinical imaging, we trained our model from scratch rather than tuning one of the provided models with initialized weights available as part of the MONAI library. We chose the UNet with transformers (UNETR) model architecture (Hatamizadeh et al., 2022). The UNETR is fully implemented in the MONAI library and is currently one of the state-of the-art model for medical image segmentation, demonstrating leading performance on the Multi Atlas Labeling Beyond the Cranial Vault (BTCV) challenge dataset for multi-organ segmentation (Hatamizadeh et al., 2022).

**Fig. 2.**
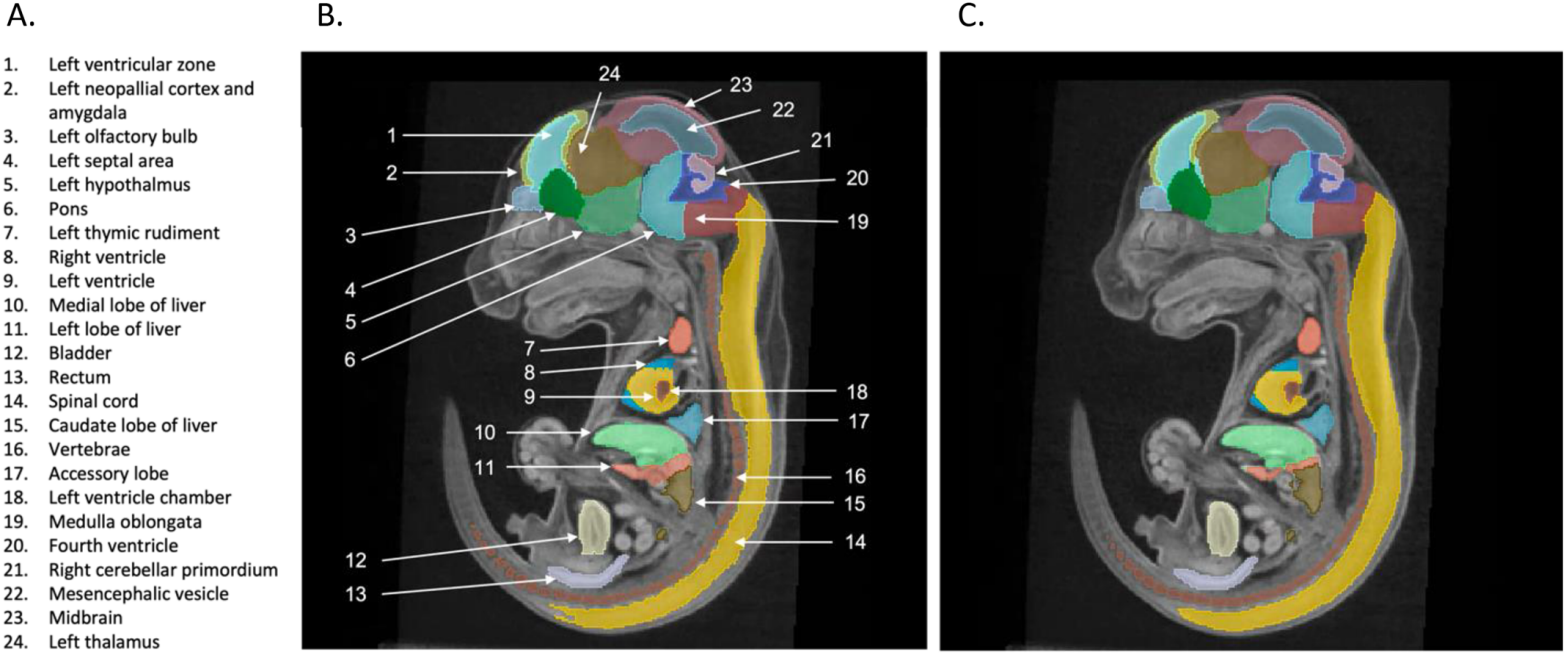
**A comparison of the (B) ABM and (C) MEMOS segmentation labels for a sagittal slice of a specimen from the test data set (not previously seen by the model for training or validation).** The 24 labels visible in this figure are defined in A. The MEMOS segmentation is visually similar, but the borders are smoother, and some segments show slight under-segmentation, including the vertebrae, accessory lobe, and the right heart ventricle.

ABM was used to generate the ‘ground-truth’ dataset to train the segmentation module. The accuracy of predicted segmentations is evaluated using the Dice coefficient of overlap between the segmentations generated via ABM and those estimated by the MEMOS model. An example of the MEMOS estimated and ‘ground truth’ segmentations for a sagittal slice of a specimen from the validation data set is shown in Fig. 2 segments. The MEMOS segmentation is visually similar for the 24 visible segments. The borders are smoother and slight under-segmentation can be seen in the vertebrae, accessory lobe, and the right heart ventricle segments.

**Fig. 3.**
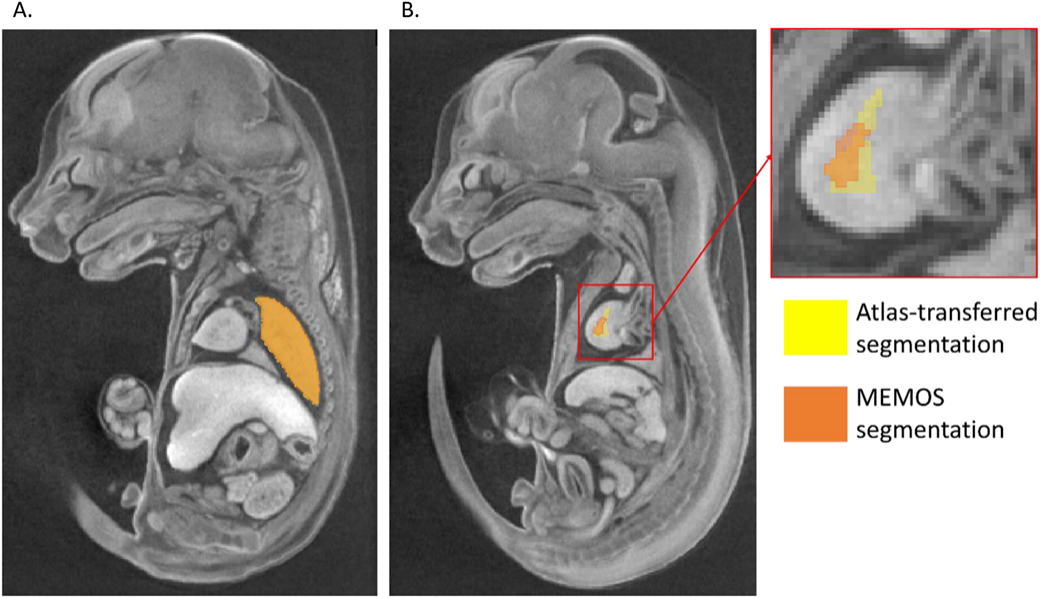
**A comparison of the ABM and MEMOS segmentations of the (A) left lung and (B) right ventricle chamber of the heart.** The left lung segment has a Dice score of 0.982162, representing one of the highest scores in the test dataset. There are very minimal differences visible at the segment border. The right ventricle chamber of the heart in the same specimen has a Dice score of 0.442308, representing one of the lowest scores. The right ventricle chamber of the heart has the poorest performance of the 50 labeled regions, due to its narrow geometry, with a width of only a few voxels at this image resolution.

The average Dice score for the fully trained model on the validation dataset is 0.89. To perform a fully objective assessment of the model effectiveness, we evaluated the performance on a test set of five specimens withheld from training and validation. For these specimens, the average Dice coefficient over all segments is 0.91, indicating similar performance on scans not used to tune the model training parameters. A breakdown of the performance of each segment in the test data set is shown in Table 1. 48 of the 50 segments have a Dice score above 0.8. The vertebrae (Dice score 0.64) and right ventricle of the heart (Dice score 0.63) have a challenging geometry at this image resolution as their width in some dimensions may be as narrow as two voxels. A comparison of ABM and MEMOS segmentations of a high performing (left lung, score=0.982162) and low performing (right ventricle of the heart, score=0.442308) segment from the same specimen in the test data set is shown in Fig. 3.

**Table 1.**
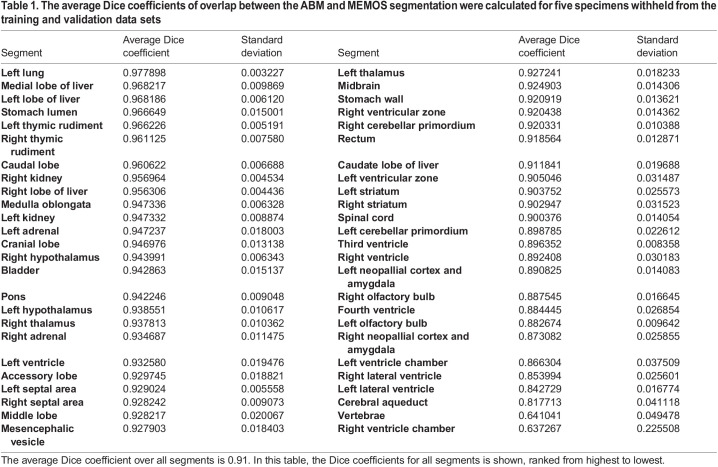
The average Dice coefficients of overlap between the ABM and MEMOS segmentation were calculated for five specimens withheld from the training and validation data sets

### Computational efficiency

To estimate the computational efficiency of the MEMOS module for segmentation, we compared the run time for the volumetric segmentation of 50 labeled anatomical structures for ten fetal mouse scans not previously seen by the model (used for training or validation) on three different compute systems. The first test system was a Linux server with an AMD Ryzen Threadripper PRO 3995WX processor with 64 cores, 512 GB RAM, and an NVIDIA A6000 GPU. The average time to place the 50 labeled regions on a single specimen was 21.9 s. Compared to the average time, as observed in our lab, for a fully manual segmentation of approximately 40 h and an ABM segmentation on the same high-powered Linux server of approximately 6 h, this represents speed-up factors of 6575 and 986, respectively. We tested the same Linux system without using the GPU, resulting in an average segmentation time for a single specimen with 50 labeled structures of 412 s, which corresponds to a speed up factor of 350 over the manual segmentation and 52 over ABM. One important feature of the MEMOS module is that it can be run on a generic desktop computer. Although this is not recommended for the most efficient use of the tool, it increases accessibility by removing the need to purchase specialized hardware. For a desktop computer with a Windows 10 Enterprise (version 21H2) operating system, 64 GB RAM, and an Intel Xeon W-2125 processor with four cores, the average segmentation time for a single specimen with 50 labeled structures was 1719.9 s (28.7 min). ABM was not run on a desktop machine for comparison since the extremely long runtime expected would make this approach impractical. The results from these experiments are summarized in Table 2.

**Table 2.**
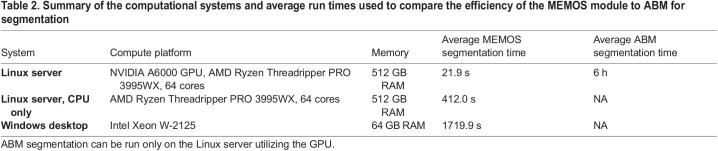
Summary of the computational systems and average run times used to compare the efficiency of the MEMOS module to ABM for segmentation

### MEMOS module

**Fig. 4.**
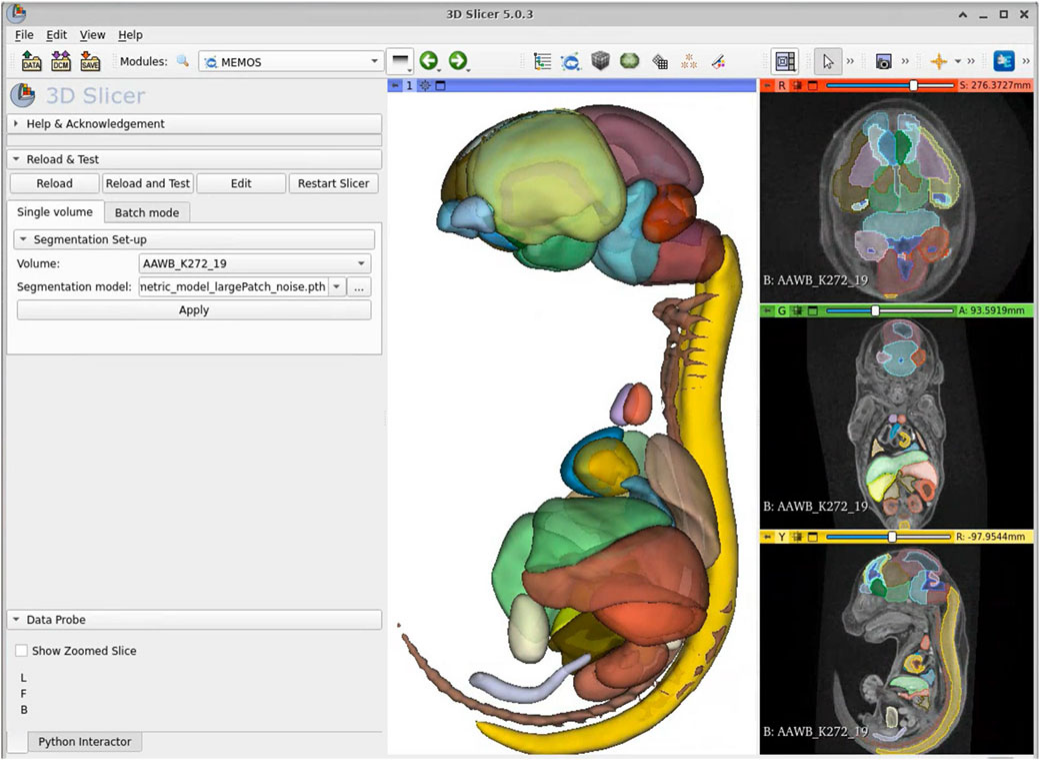
**The MEMOS module interface deployed via the 3D Slicer application for segmentation of new specimens.** The 3D Slicer application provides interactive visualization, editing, and statistical analysis of the segments.

Our trained segmentation model is deployed in the MEMOS module that is available as an extension of the 3D Slicer platform. 3D Slicer is an opensource software package that leverages powerful image analysis libraries such as Visualization Toolkit (VTK), and Insight Toolkit (ITK), and an increasing number of state-of-the-art Python packages including the MONAI library to support common image analysis tasks and the creation of custom extensions and workflows (Fedorov et al., 2012; Kikinis et al., 2014). The module estimates segmentation of new images in an application providing easy access to tools to review and edit the labels and requiring no programming expertise. The MEMOS module interface is shown in Fig. 4. The supplementary data that accompanies the paper provides the necessary steps to install and use the MEMOS module.

### Segmentation of knockout mutants with abnormal anatomy

**Fig. 5.**
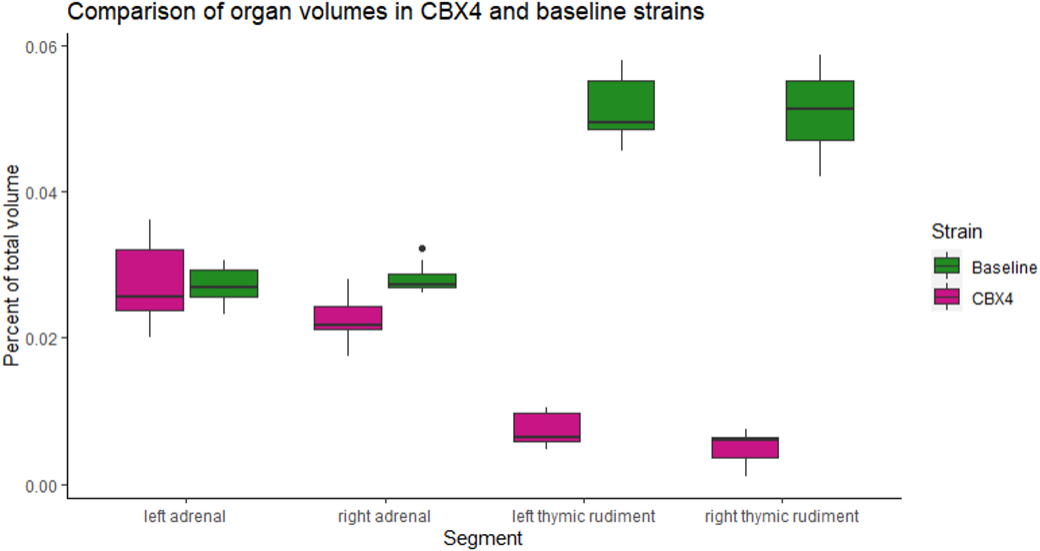
**Volumetric comparison of the baseline (*n*=11) and *Cbx4* knockout (*n*=8) strains for the left and right adrenals and the left and right thymic rudiments.** The volume difference between the strains is a statistically significant for the right adrenal (*P*=0.001), left thymic rudiment (*P*<0.001) and right thymic rudiment (*P*<0.001). The difference is not statistically significant for the left adrenal (*P*=0.985).

**Fig. 6.**
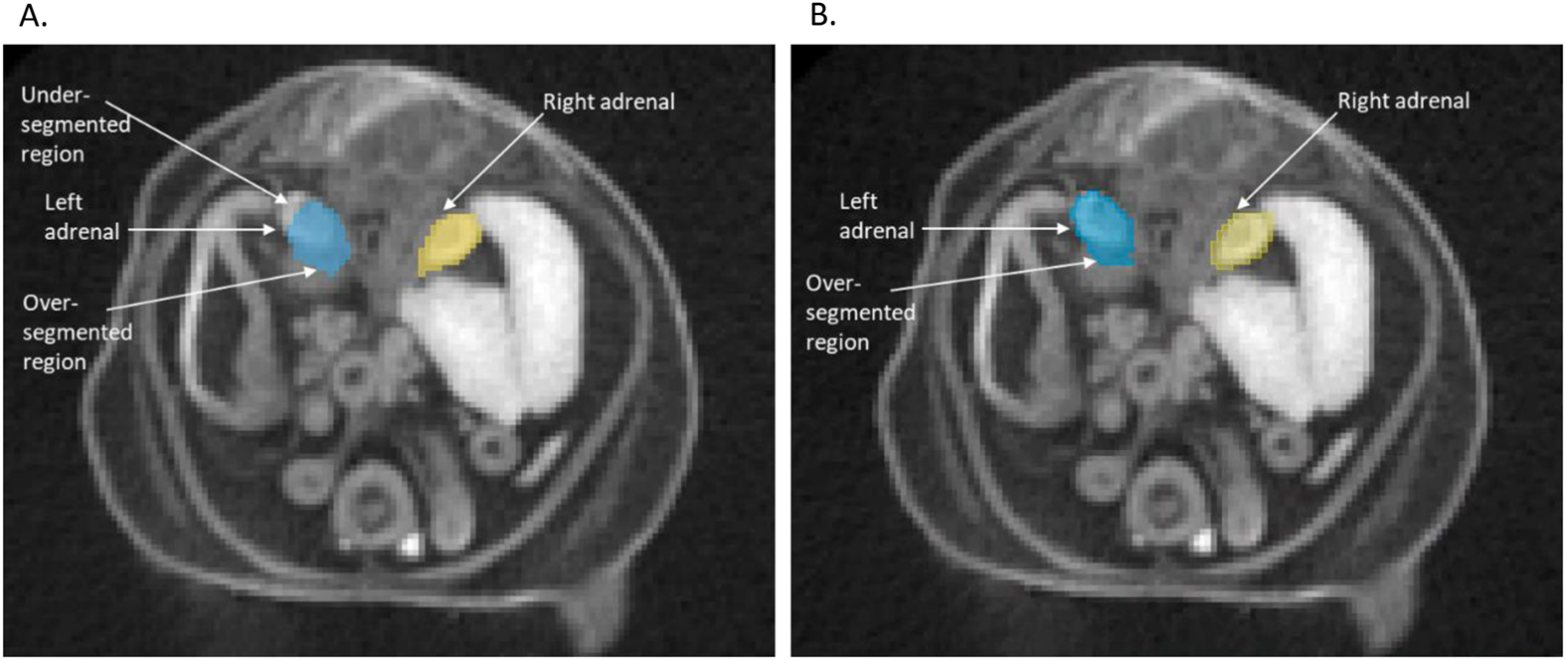
**Axial slice from a *Cbx4* knockout mutant showing (A) ABM and (B) MEMOS segmentation labels for a region with known anatomical abnormality.** The ABM segmentation has both under and over-segmented regions of the left adrenal gland (Dice score of 0.591 with the manual segment). The MEMOS segmentation shows over-segmentation of the left adrenal gland (Dice score 0.818 with the manual segment).

As the primary application of this segmentation tool is to investigate abnormal morphology in mouse models, the ability to detect small-scale differences in segment size and shape is a key performance metric for MEMOS. To test the segmentation sensitivity, we chose the *Cbx4* knockout strain from the KOMP dataset, with a phenotype that has been previously reported to have a statistically significant reduction in volume of the left and right thymic rudiment and the left and right adrenals (Dickinson et al., 2016; Liu et al., 2013). We used the MEMOS module to segment homozygous specimen from the knockout *Cbx4* strain (*n*=8) and compared the adrenal and thymic rudiment volumes, as a percentage of whole-body volume, to those in a test dataset of images not previously seen by the model (*n*=11). The differences between the segment volumes of the two strains segment were determined by a Student’s *t-*test with a significance threshold of 0.05. As detailed in Fig. 5, there is a statistically significant decrease in volume of the right adrenal (*P*=0.001), left thymic rudiment (*P*<0.001) and right thymic rudiment (*P*<0.001) in the *Cbx4* knockout strain group. The left adrenal also has a lower average volume, though the difference was not statistically significant (*P*=0.985). An example of the adrenal segmentation of a *Cbx4* knockout specimen using (A) ABM and (B) MEMOS is shown in Fig. 6. Both segmentations show regions of over or under-segmentation in the left adrenal. In this specimen, the MEMOS segmentation is closer to the manual segmentation (Dice score of 0.818) than the atlas transferred segmentation (Dice score of 0.591). Both automated segmentations may be subject to bias towards normal anatomy that can cause errors when segmenting mutants. For ABM this may be introduced by limitations of the deformable transformation and the selection of an atlas generated from only baseline subjects. In the case of MEMOS, this bias may be introduced using baseline specimens only for training.

## DISCUSSION

In this work we have introduced MEMOS, a fully open-source, deep learning-enabled tool for automated estimation of multi-organ segmentations from fetal mouse scans. MEMOS was developed with the goal of supporting the segmentation of imaging data collected as part of the NIH Common Fund KOMP2 project. When using a high-performance server, 50 anatomical structures can be estimated within 2 min. MEMOS can also provide meaningful time savings with no specialized hardware. The accuracy of MEMOS-generated segmentations is comparable to gold-standard methods that require multiple orders of magnitude more computation time and significant computational resources. The sensitivity of this tool supports its use with the KOMP2 database on both baseline specimens and strains with known anatomical abnormalities.

The MEMOS segmentations can be generated, reviewed, and if necessary, edited within a single open-source application, creating a highly streamlined platform for semi-automated segmentation. The 3D Slicer application provides both the platform for the MEMOS module and an extensive and mature set of interactive segmentation tools that can be applied through the user interface or automatically through simple Python scripts. For example, the MEMOS module can produce small regions of over-segmentation that are spatially disconnected from the remainder of the segmentation. The Segment Editor module within the 3D Slicer application provides an ‘Islands’ tool that identifies the connected components of a segment and can be used to remove small, disconnected regions with a single button click. The combination of fast initial segmentations produced by MEMOS and accessible, streamlined segment editing tools can be used to quickly generate segmentations for analysis with accuracy that exceeds the MEMOS experimental results.

### Customizing the MEMOS deep learning-model

While goal of this project is to introduce a general-purpose tool for fetal mouse segmentation, the MEMOS deep learning-model can also be customized for specific segmentation tasks. Currently, there are no pre-trained models available for fetal mouse segmentation, so the MEMOS segmentation model provides an important resource for applications where similar datasets are being analyzed. Customizing our model will require Python programming experience, installation of opensource software packages and access to a deep-learning capable server to retrain the MEMOS model. The retrained model can then be loaded and used for segmentation through the existing MEMOS module in 3D Slicer. Here, we briefly address three use cases that may benefit from additional model training.

#### Less-supervised multi-organ segmentation

While the ABM method for segmentation used to generate our training and validation labels is currently state-of-the-art, it can also be a source of segmentation error. Deep learning models do not have the same limits on morphological variability that can be captured by ABM, so performance of our segmentation model can be improved by training on test data that was segmented via the MEMOS model and manually edited to correct misplaced ABM labels. To further improve the tolerance of MEMOS for high levels of abnormality such as can be present in the KOMP2 knockout strains, additional specimen with anatomical abnormality could be added to the training set to model a broader range of local shape variation. Increasing the accuracy of the MEMOS module through these strategies can reduce the need for manual editing, though a fully automated segmentation will likely require advances GPU memory available.

#### Segmentation of a single or subset of organs

Solving the problem of whole-body segmentation is much more complex than a single segment, or smaller number of segments. For applications where the full set of 50 labels is not necessary, MEMOS could be tuned for segments of interest. To tune MEMOS to a single organ, the model can be further trained on cropped patches centered on that organ. These additional examples can increase the accuracy of the model for that segment, while reducing the burden of editing new training segmentations, since fewer segments need to be placed.

#### Segmentation of outside datasets

While MEMOS was trained exclusively on data from the KOMP2 database, our model can be retrained with E15.5 fetal mouse scans from other sources, such as microCT scans acquired at institutions using different scan settings or imaging protocols, to generate a customized segmentation model for a specific dataset. With adequate training data, this model could be modified to create a segmentation model for other developmental stages, though this likely be most useful for neighboring stages where the segments have structural similarity.

### Use of ABM segmentation for model training

Manually segmenting hundreds of datasets with 50 segments each would have taken tens of thousands of person-hours, which was simply not possible for this project. Due to practical constraints such as these on the time and labor required by manual segmentation, ABM has been the gold-standard approach in large-data applications for over a decade (Baiker et al., 2010; Wong et al., 2012). In addition to being impractical, manual segmentation is also ultimately a subjective task, prone to human bias and error, as even experts can disagree considerably in their manual labelling of anatomy, a fact that is well documented in medical imaging literature (Zou et al., 2004; Norris et al., 2013; Heijman et al., 2008; Chlebus et al., 2019).

**Fig. 7.**
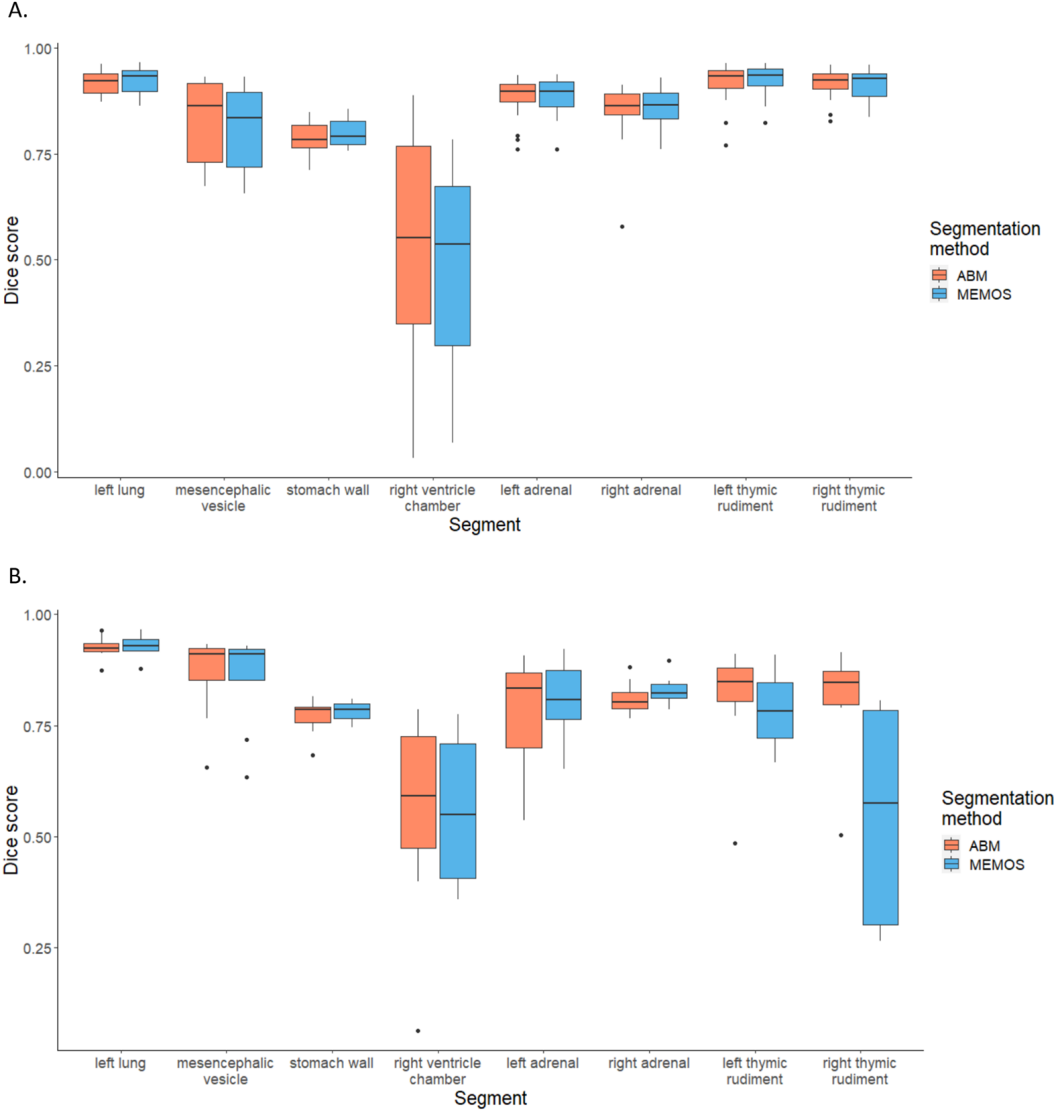
**Comparisons of ABM and MEMOS-generated segmentations to labels placed manually by an expert.** (A) Dice scores for the baseline strain (*n*=22). (B) Dice scores for the *Cbx4* knockout strain (*n*=8). There is no significant difference between the ABM and MEMOS segmentation Dice scores for the subset of segments, with the exception of the right thymic rudiment in the *Cbx4* knockout strain (*P*=0.021).

While we propose MEMOS as a replacement for the widely accepted ABM approaches frequently used in big-data applications rather than manual segmentation, it is useful to understanding the performance differences between the automated methods. To ABM and MEMOS to manual segmentation, we segmented eight structures for a subset of 22 baseline and eight *Cbx4* knockout strain specimens, using each of the three methods. The eight segments were selected to include high, average, and low preforming segments and the four segments known to have anatomical abnormality in the *Cbx4* knockout strain. Analysis of Dice overlap between each automated method and the manual segmentation is detailed in Fig. 7. For the baseline specimens, there was no significant difference between the automated methods for any of the eight segments. For the *Cbx4* knockout specimen, only the right thymic rudiment in the knockout strain showed a statistically significant difference (*P*=0.021). In this case, ABM was more similar to the manual segmentation. It should be noted that this difference in the MEMOS segmentation did not prevent detection of a volumetric difference in this segment, as shown in Fig. 5. The results of this experiment provide confidence that (1) the ABM method produces comparable results to manual segmentation and (2) MEMOS provides a competitive alternative to the computationally expensive ABM segmentation. The segmentation of the right thymic element also demonstrates a case in which using MEMOS alone can flag the presence of abnormality, but manual editing would be recommended before quantitative analysis of volumetric differences.

### Hardware limitations

Currently, the resolution of the MEMOS segmentation network is constrained by the hardware available for deep learning. While state-of-the-art equipment was used to train the UNETR model, the available memory on current GPUs was not sufficient to train on the images at the original resolution. One way to address this constraint is to downsample the images to a resolution that can be directly input into the network. However, the interpolation operations during downsampling can create indistinct boundaries between anatomical regions, breaking biological structure. An alternative strategy that preserves the content of the scans is to break the volume into smaller regions that are used as individual training examples. For applications with a standard space or low variation among subjects, regular patches and their coordinates could be used to preserve positional information lost in sampling. However, full-body segmentation that includes highly plastic abdominal organs have no such expectation of spatial regularity and so we have employed a strategy of sampling randomly selected, overlapping regions. The region size chosen is near the maximum size allowable by our GPU server to preserve spatial context from the volume. In the future, with improved hardware available, the model could be retrained on images at the full resolution to improve segmentation accuracy, towards the goal of fully automated segmentation.

The MEMOS module provides a highly accessible, efficient, semi-automated segmentation pipeline by combining the benefits of a deep learning-enabled segmentation estimates with expert knowledge input via an intuitive interface. MEMOS can produce fast, high-quality segmentations with a minimal amount of user interaction and no requirements for specialized hardware. As the first publicly available pre-trained model for fetal mouse segmentation, we believe the MEMOS deep learning model will be an important contribution to many research applications using fetal mouse imaging.

## MATERIALS AND METHODS

### Imaging data

As part of the IMPC KOMP2 protocol, iodine contrast-enhanced whole-body micro-CT scans are collected at embryonic stage 15.5 from baseline and knockout strains that are lethal or sub-viable (Dickinson et al., 2016). A consensus population average image for stage E15.5 has been previously calculated from 35 C57BL/6 baseline specimens (Dickinson et al., 2016; Wong et al., 2012). This average image was manually segmented with 50 labeled anatomical regions in a process taking approximately 400 h to ensure accuracy (Wong et al., 2012). Both the consensus population average image and segmentation are publicly available as part of the KOMP2 project and were used in this project to generate the MEMOS training dataset via ABM. The baseline and *Cbx4* knockout specimen scans were obtained via the IMPC web portal. Due to memory constraints on currently available hardware for deep learning, we used the low-resolution release of the scan data. The scans have variable sizes, with approximately 250×250×400 voxels each.

### Generating training and validation data

To generate the segmentations required for training and validation, 91 baseline scans from the KOMP2 dataset were aligned rigidly and then deformably to the KOMP2-provided atlas image, in a two-step process implemented using open–source advanced normalization tools (ANTs) image quantification library, and its associated R library (ANTsR) (Avants et al., 2014; Tustison et al., 2021). After the registration, the inverse of the transformation between the atlas and individual is used to map the atlas segmentation into the space of each individual specimen.

### Model architecture

Our model utilizes a UNETR architecture as described in Hatamizadeh et al. (2022) with encoding and decoding units arranged in a UNet-like contracting-expanding pattern. The 3D input volume is divided into uniform, non-overlapping patches that are flattened into 1D sequences. These sequences are projected via a linear layer into a *K*-dimensional embedding space. A 1-dimensional learnable positional embedding is added to preserve the spatial relationship of the patches. The encoding path consists of a stack of transformers that learn the sequence representations of the input volume. At the bottleneck of the transformer encoder, a deconvolutional layer is used to increase the feature map resolution by a factor of 2. The outputs of the encoding layers are merged with a decoder via skip connections at various resolutions. The decoding units consist of a consecutive 3×3×3 convolution followed by up-sampling through a deconvolutional layer. At the final decoding layer, the output has the original input resolution and is fed into a 1×1×1 convolutional layer that uses a softmax activation function to generate predictions for each voxel in the original volume. The loss function computes both Dice and cross-entropy loss and uses an equally weighted combination.

### Model implementation details

Our UNETR model was implemented using the MONAI and Pytorch on a server with 512 GB RAM and A6000 GPUs. The model was trained on the training dataset for 80,000 iterations, using the AdamW optimizer with an initial learning rate of 0.0001. Inference on the validation dataset was reported at increments of 500 iterations. The transformer encoder was implemented with 12 layers, and an embedding dimension size of *K*=768. The non-overlapping patch resolution used was 16. Inference of the segmentation predictions was done using a sliding window approach on the full-resolution testing and validation images. The inference window size is 128×128×128 with an overlap factor of 0.8 between the windows.

### Model training

The input volumes are randomly sampled with a volume of 128×128×128 voxels, and the intensity is normalized. The subsampled volumes are augmented by random rotation, affine transformation, intensity shifting, and addition of Gaussian noise. The training and evaluation strategy was set up to assess how well the MEMOS model could generalize to unseen scans. Towards this goal, we divided the scans into three sets, with 73 images used for training of the model weights, 18 images used to validate the model hyperparameters, and five scans withheld for testing.

## Supplementary Material

10.1242/biolopen.059698_sup1 Supplementary information
