# Supplementary material for: Deep learning enabled multi-organ segmentation of mouse embryos
Source: Biol Open. 2023 Feb 21;12(2):bio059698. doi: 10.1242/bio.059698 (PMC9990908; doi:10.1242/bio.059698)
Supplement: Supplementary information [file biolopen-12-059698-s1.pdf]

Supplementary Information

Installing and running the MEMOS module.

The MEMOS module can be run on Windows, Mac, or Linux OS, on any standard desktop. However, to achieve the maximum benefit to segmentation speed reported in this work, the module should be run on Windows or Linux machine with a CUDA-capable GPU.

1. Download and install 3D Slicer from https://www.slicer.org. Please use the stable release (version 5.2.1) for the most dependable results.

2. Download the latest trained MEMOS deep learning model from the following link: https://app.box.com/shared/static/4nygg33o70oj5xvnhew11zz5geclus5b.pth

3. **Optional Setup for GPU accelerated inference:** To benefit from GPU acceleration, your computer needs to be setup with the CUDA development kit library for your OS and compute platform and a compatible version of PyTorch. If this is skipped, the default version of PyTorch that supports only CPU processing will be installed with the MEMOS module. These steps require an internet connection and will require restarting the 3D Slicer application.
    a) Start the 3D Slicer application and open the extension manager. Switch to the “Install Extensions” tab. Search for and install the PyTorch utilities extension. Restart the 3D Slicer application to activate the “PyTorych Utils” module.

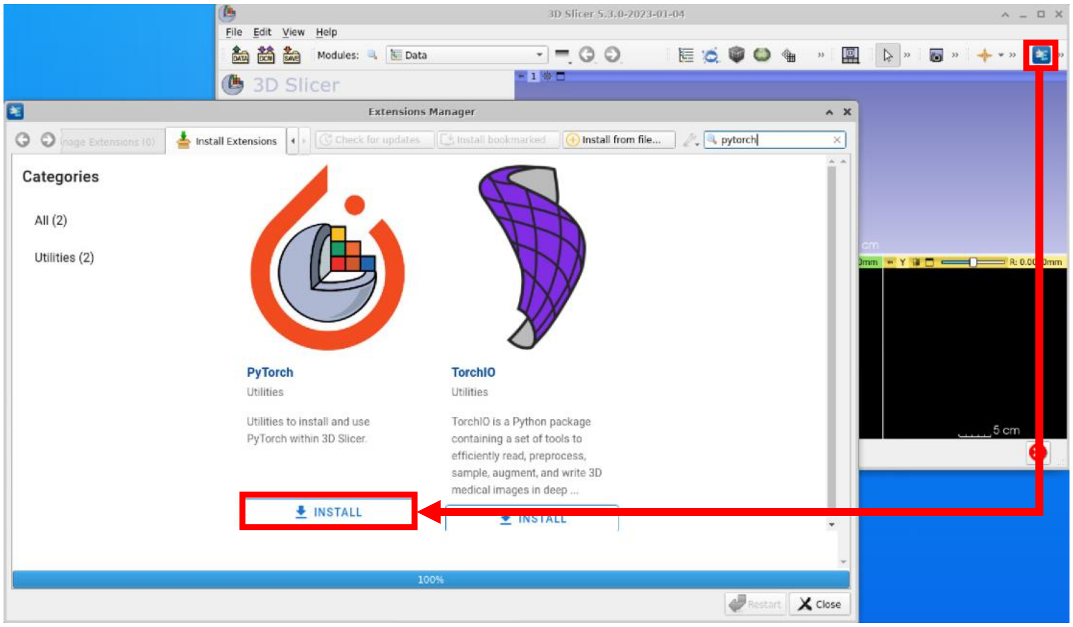

Biology Open • Supplementary information

b) Open the “PyTorych Utils” module in 3D Slicer. Check the PyTorch field at the top of the module. If PyTorch has been previously installed, it can be uninstalled first by clicking the “Uninstall PyTorch” to ensure the correct version will be obtained for your computing platform.

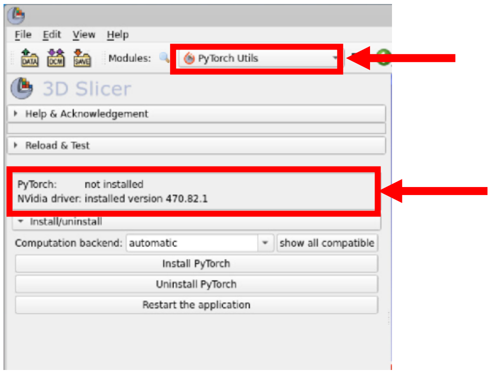

c) Click “Install PyTorch” from the “Install/uninstall” menu to start the installation. This step may take a couple of minutes. This module automatically detects the most appropriate computational backend given the available hardware. To make a different selection, click “show all compatible” and chose from the “Computational backend” drop-down menu.

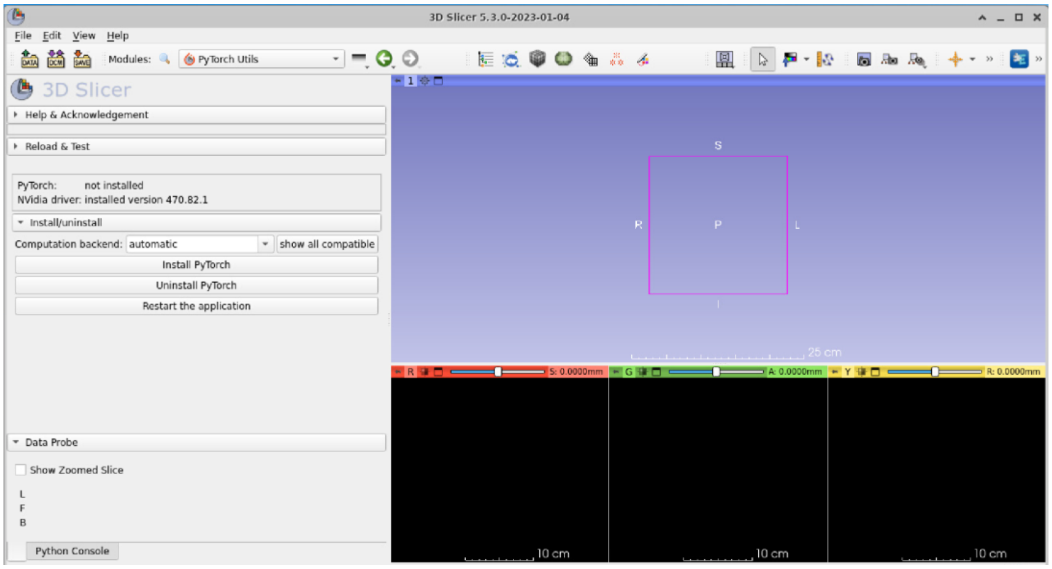

Alternatively, CUDA and PyTorch can be installed manually. For details, please see https://pytorch.org/get-started/locally/.

4. **Install the MEMOS extension in 3D Slicer:** The MEMOS extension can be downloaded and installed from the 3D Slicer application. For additional documentation of the

MEMOS extension please see https://github.com/SlicerMorph/SlicerMEMOS. The following steps require an internet connection and restarting the 3D Slicer application.

a. Click on the Extension Manager icon in the upper toolbar of the 3D Slicer application.

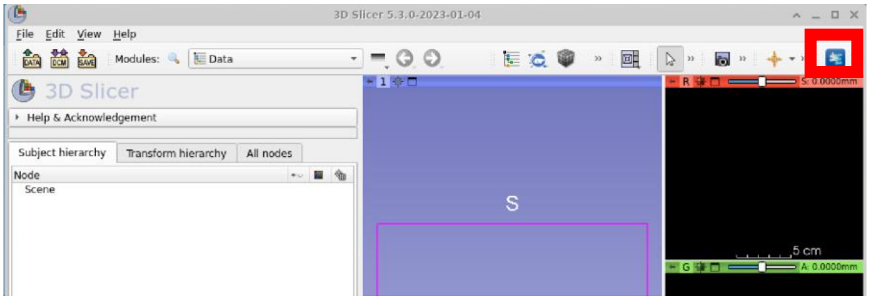

b. The Extension Manager will be displayed as a pop-up window. Select the "Install Extensions" tab at the top of this window. All the available extensions will be displayed. Search for "MEMOS" using the search bar at the top of the window.

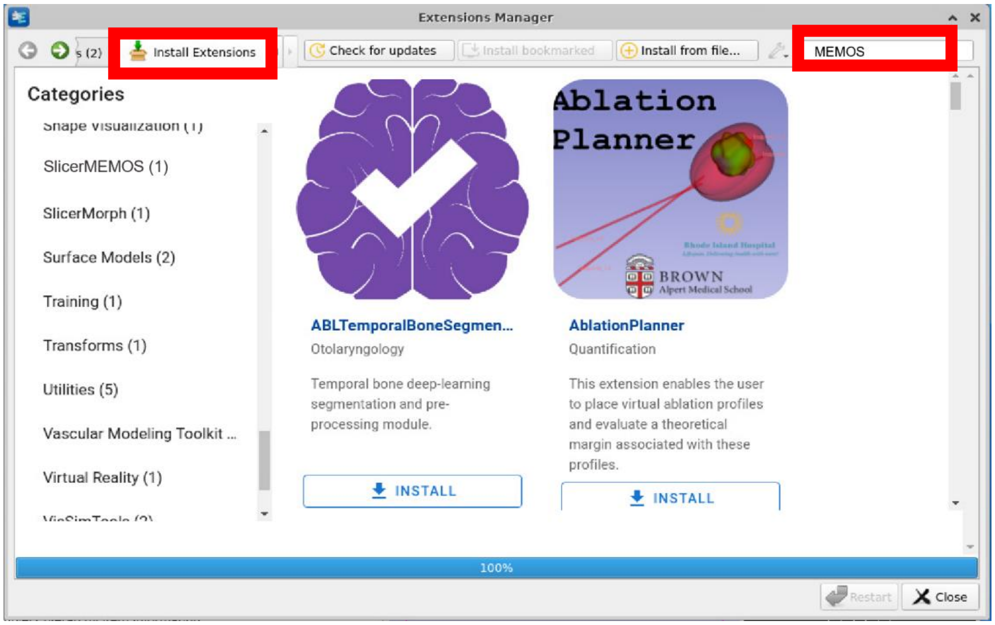

c. Click the "Install" button under the MEMOS icon to download and install the MEMOS extension. The application will need to be restarted to access the module. This can be done using the "Restart" button in the lower right corner of the Extension Manager, or by closing and reopening the 3D Slicer application.

Biology Open • Supplementary information

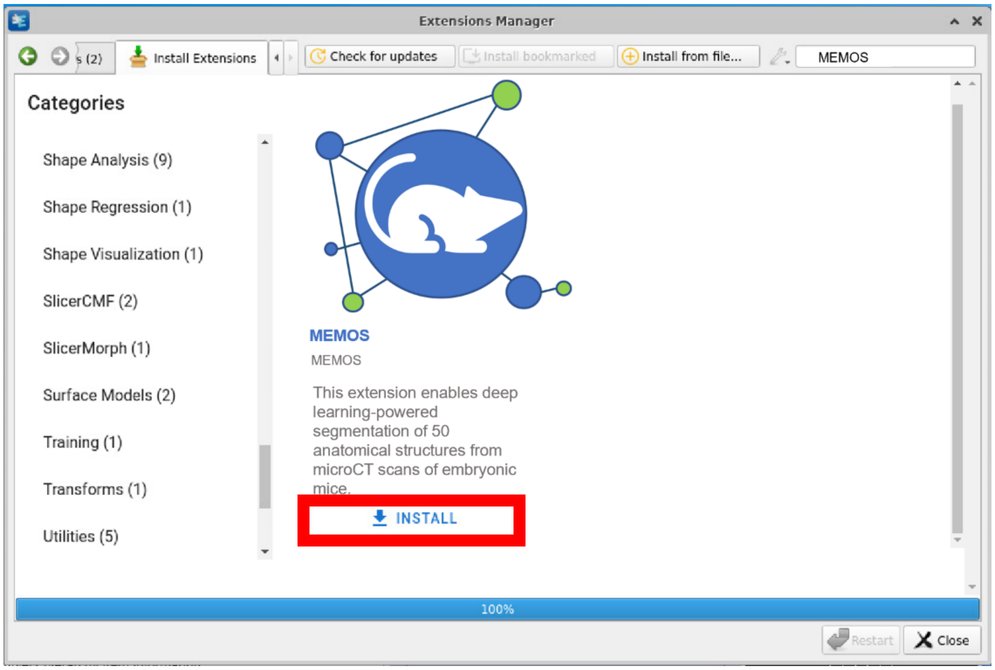

5. **Open the MEMOS module:** When opening the module for the first time, the application will check for required Python libraries, including Pytorch, MONAI, and their dependencies. Any libraries not found in the Slicer version of Python will be installed at this time. This step will require an internet connection and may take a couple minutes.

6. **Segmenting a single volume in the scene:** The MEMOS module has two modes for segmentation: single volume, where one image is loaded into the Slicer application and segmented; and batch mode, where all images in a folder are segmented and the results are saved to specified output folder.

    a. To run MEMOS on a single volume, load the volume into the Slicer application, by dragging and dropping the file into the scene, or using the 3D Slicer "Add Data" dialog box.

    b. In the MEMOS module, check that the "Single volume" tab is selected. In the "Volume" field, select the loaded image from the drop-down menu. In the "Segmentation model" field, browse to the MEMOS deep learning model downloaded in step 2. When these fields have been filled in, click "Apply". MEMOS will estimate a segmentation for the volume and once it is complete, it will be displayed in the scene. (Note: If you are not using the GPU acceleration this can take anywhere from 20 minutes to an hour and dependent on the CPU power available in your computer)

Biology Open • Supplementary information

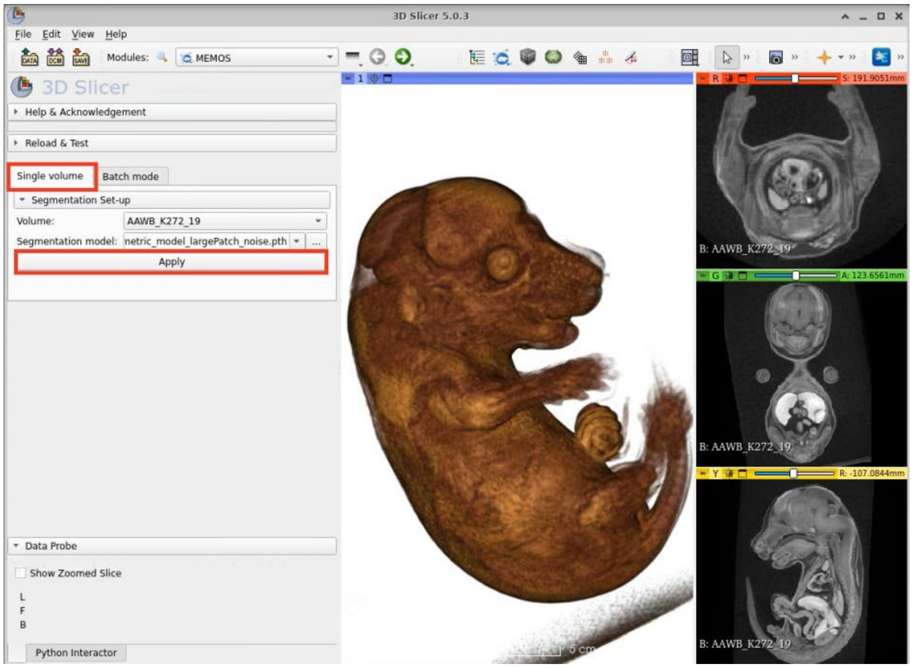

7. **Manual Refinement:** To review and edit the segmentation, browse to the Segment Editor module. For a detailed guide on using the tools in the Segment Editor please see the official user guide: https://slicer.readthedocs.io/en/latest/user_guide/modules/segmenteditor.html and tutorials developed by our research group here: https://github.com/SlicerMorph/Tutorials/blob/main/Segmentation/Segmentation.md

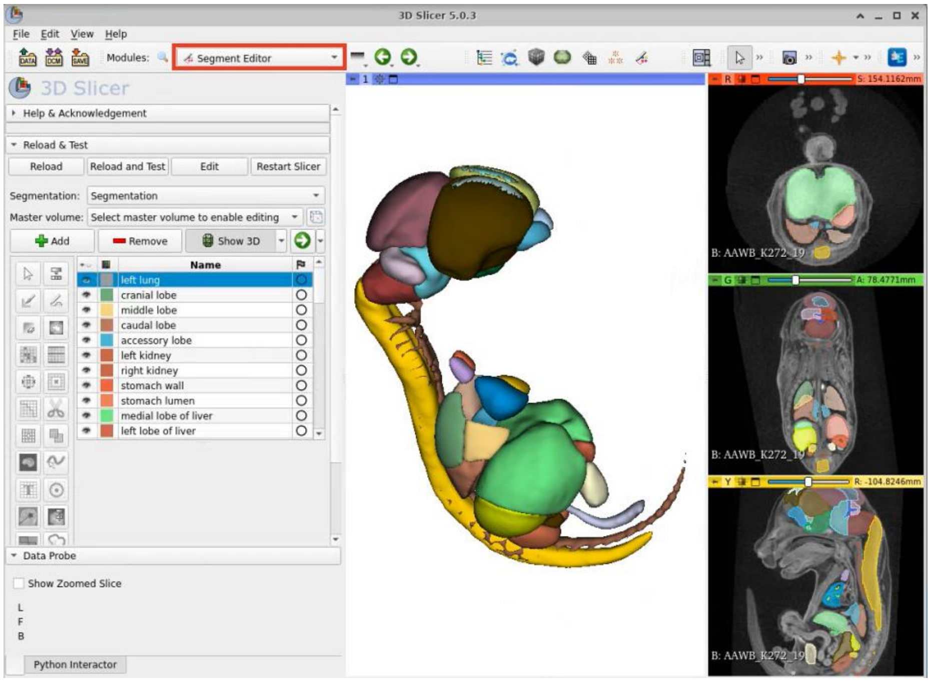

Biology Open • Supplementary information

8. **Save finalized segmentation:** Once the segmentation has been reviewed and edited, it can be saved to file using the 3D Slicer "Save Data" dialog box.

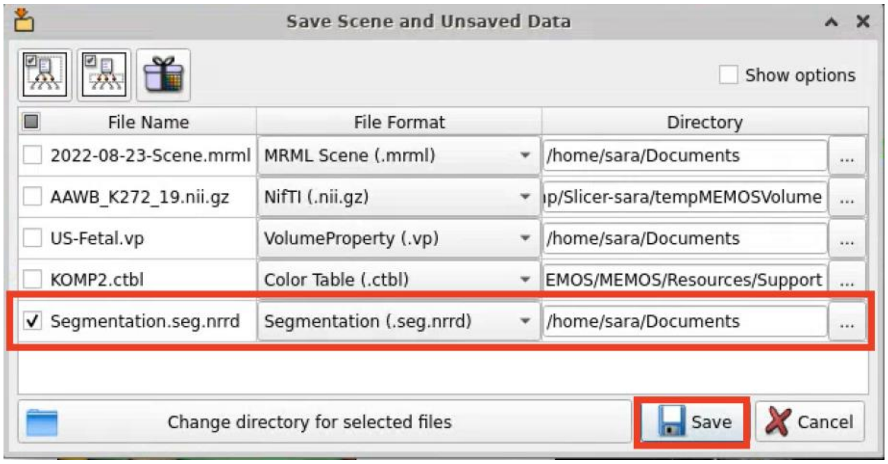

9. **Segmenting volumes in batch mode:** To run MEMOS on each volume in a folder and save the output segmentations, switch to the "Batch mode" tab of the module. Select the folder where the volumes are saved in the "Volume directory" field. In the "Segmentation model" field, browse to the MEMOS deep learning model downloaded in step 2. Select the folder where the segmentations for each volume will be saved in the "Output directory field". Click "Apply" to generate the segmentations.

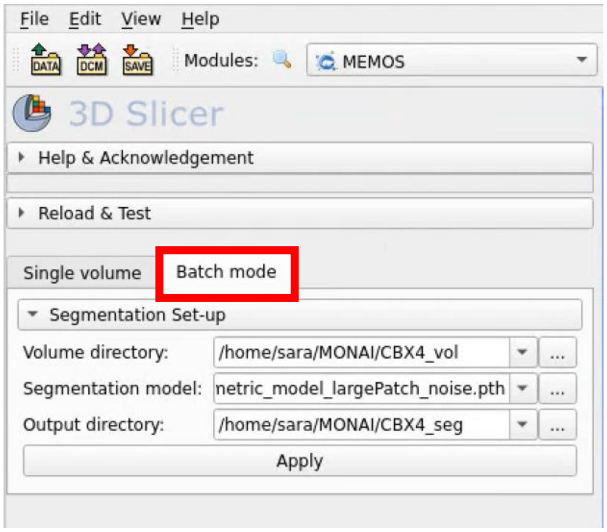

Biology Open • Supplementary information

10. **Manual refinement of batched segmentations:** To review and edit the segmentations produced in batch mode, load them from the selected output file into the 3D Slicer scene as a segmentation and open the Segmentations Editor module.

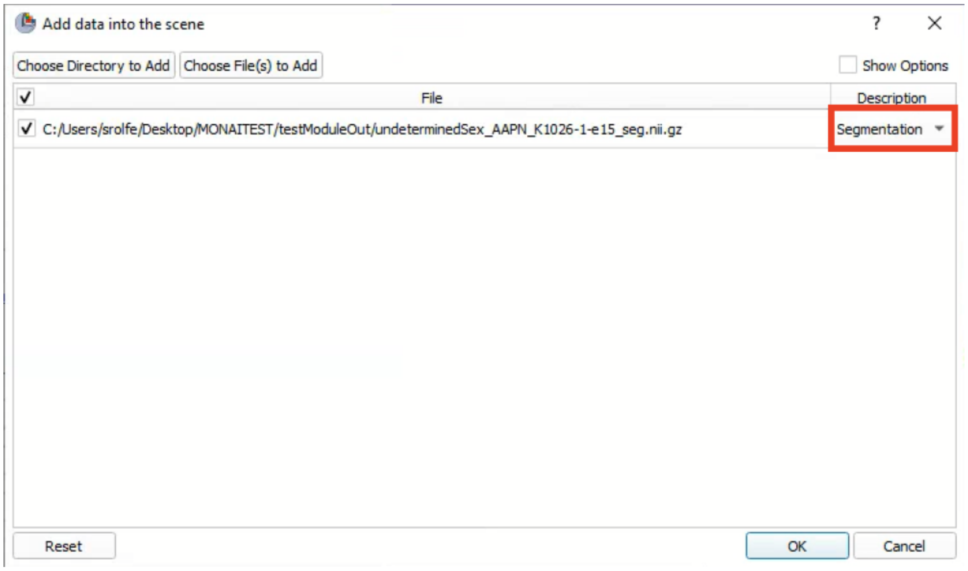

Biology Open • Supplementary information
